# Ocular Allergy Modulation to Hi-Dose Antigen Sensitization Is a Treg-Dependent Process

**DOI:** 10.1371/journal.pone.0075769

**Published:** 2013-09-27

**Authors:** Hyun Soo Lee, Simona Schlereth, Payal Khandelwal, Daniel R. Saban

**Affiliations:** 1 Department of Ophthalmology, Duke University School of Medicine, Durham, North Carolina, United States of America; 2 Department of Immunology, Duke University School of Medicine, Durham, North Carolina, United States of America; 3 Department of Ophthalmology, Schepens Eye Research Institute, Massachusetts Eye and Ear Infirmary, Harvard Medical School, Boston, Massachusetts, United States of America; Research Center Borstel, Germany

## Abstract

A reproducible method to inhibit allergic immune responses is accomplished with hi-dose Ag sensitization, via intraperitoneal (IP) injection. However, the role of CD4+ CD25+ FoxP3+ T regulatory cells (Treg) in this process is unknown, as is whether such modulation extends to ocular allergy. We therefore determined herein whether hi-dose sensitization modulates ocular allergy, and whether CD4+ CD25+ FoxP3+ Treg are involved. C57BL/6 mice were IP sensitized via low-dose (100 µg) versus hi-dose (1000 µg) ovalbumin (OVA), in aluminum hydroxide (1 mg) and pertussis-toxin (300 ng). Other mice received anti-CD25 Ab (PC61) to ablate Treg during sensitization. In another experiment, Treg from hi-dose sensitized mice were adoptively transferred into low-dose sensitized mice. Once daily OVA challenges were administered. Clinical signs, IgE, T cell cytokines, and eosinophils were assessed. Data revealed that hi-dose, but not low-dose, sensitization led to allergy modulation, indicated by decreased clinical signs, serum IgE levels, Th2 recall responses, and eosinophil recruitment. T cells from hi-dose sensitized mice showed a robust increase in TGF-b production, and Treg from these mice were able to efficiently suppress effector T cell proliferation in vitro. In addition, in vivo Treg ablation in hi-dose sensitized mice revoked allergy modulation. Lastly, Treg from hi-dose sensitized mice were able to adoptively transfer allergy modulation to their low-dose sensitized counterparts. Collectively, these findings indicate that modulation to hi-dose sensitization, which is extended to ocular allergy, occurs in a Treg-dependent manner. In addition, our data suggest that hi-dose sensitization may henceforth facilitate the further examination of CD4+ CD25+ FoxP3+ Treg in allergic disease.

## Introduction

The biology of regulatory T cells (Treg) continues to be an active area in allergy research and a focus to help further understand potential contribution(s) to normal allergy resistance and therapy. These naturally occurring immuno-modulatory lymphocytes are of tremendous interest to the field because of their crucial role in the maintenance of normal immune homeostasis. Evidence for Treg in staving off autoimmune diseases is widely appreciated, and likewise there is literature supporting their role in modulation of T helper 2 (Th2) responses that mediate allergy [1]. Two main populations of Treg which have received considerable attention, include (a. CD4+ CD25+ FoxP3+ Treg; and (b. CD4+ IL-10 secreting Treg (i.e. Tr1) [1,2].

Despite significant advances in this area, key aspects, such as in FoxP3+ Treg function in allergy, remain incompletely understood. For example, on the one hand, FoxP3+ Treg do not appear to be involved in allergy regulation that occurs in healthy individuals. Evidence for this was shown by Meiler et al, as protection to hi-dose bee venom exposure in beekeepers was mediated instead by Tr1 [3]. On the other hand, disruption of FoxP3+ Treg is known to lead to dysregulation of allergic immune responses, as evidenced to Foxp3 mutations in scurfy mice and in humans (i.e. immune dysfunction, polyendocrinopathy, enteropathy, X-linked syndrome) [4,5]. Thus, future work, which includes further development of incisive animal models, continues to be a crucial component to delineate such disparate Treg mechanisms, such as in allergy modulation versus susceptibility.

HI-dose intraperitoneal sensitization in mice is a highly reproducible model to induce an impairment of allergic Th2 responses [6-12], and thereby is of particular utility toward elucidation of natural mechanisms that modulate allergy. Evidence has led to the hypothesis that such an altered response is merely a secondary effect of priming a Th1 dominant response—which in turn suppresses other Th subsets such as Th2 [6-8]. However, there are a number of studies that have challenged this dogma with the observation that an increased Th1 response was not observed in response to hi-dose intraperitoneal sensitization [9-12]. Furthermore, it is now widely appreciated that allergic immune responses are contributed to by other Th subsets such as Th1 and Th17 [13-17]. Collectively, these reports point to the notion that a Th1 dominant suppression cannot fully explain the allergy modulation that arises from hi-dose intraperitoneal sensitization.

Data from the current study show that Treg are central to modulating ocular allergy in hi-dose intraperitoneal sensitization. This is supported by our findings that Treg ablation revokes allergy modulation afforded by hi-dose intraperitoneal sensitization, and we also show that Treg from hi-dose sensitized mice adoptively transfers this modulatory effect. Thus, we conclude that Treg mediated tolerance is responsible for the modulation of allergy responses arising from hi-dose intraperitoneal sensitization. Collectively, our findings reveal an important mechanism by which hi-dose intraperitoneal sensitization modulates Th2 in allergy, and thus support the use of hi-dose intraperitoneal sensitization as a model for future studies of CD4+ CD25+ FoxP3+ Treg biology and tolerance mechanisms relevant in allergy.

## Materials and Methods

### Animals and anesthesia

Eight to 10 week-old male C57BL/6 mice were purchased from Charles River Laboratories (MA). Mice were kept in a specific pathogen-free environment at the animal facility of Schepens Eye Research Institute. The Institutional Animal Care and Use Committee at Schepens Eye Research Institute approved the experimental protocol, and all animals were managed according to the ARVO Statement for the Use of Animals in Ophthalmic and Vision Research. Anesthesia was used for all surgical procedures with intraperitoneal administered ketamine/xylazine suspensions (120 and 20 mg/kg, respectively). Euthanasia was performed in accordance with American Veterinary Medical Association Guidelines for Euthanasia of Animals. This was accomplished via carbon dioxide asphyxiation with a precharged chamber, followed by a secondary measure to ensure non-recovery accomplished by bilateral thoracotomy.

### Sensitization and ocular surface challenge to induce ocular allergy

This model has been previously described [17]. Briefly, C57BL/6 mice were sensitized via intraperitoneal injection once with low-dose (100µg) or hi-dose (1000 µg) ovalbumin (OVA, Sigma Aldrich). Injection included 1 mg aluminum hydroxide (Sigma Aldrich), and 300 ng of pertussis toxin (Sigma Aldrich) diluted in 100 µl PBS. Mice were rested for 2 wks and then challenged by instillation of OVA eye drops (250 µg/5 µl) once daily for at least 12 d.

### Clinical scoring

This procedure has been previously described [17]. Briefly, clinical evaluation of ocular allergy was performed in a masked fashion for signs of immediate hypersensitivity responses 20 min after topical OVA challenge. Clinical evaluation was based on four parameters, which included: lid swelling, tearing, conjunctival chemosis, and conjunctival redness. The severity of each parameter was graded from 0 (absent of signs) to 3+ (maximal) scale and the total score consisted of the sum of these four parameters.

### ELISA for sera OVA-specific IgE

Following topical challenge, blood was collected from submandibular vein of mice and sera were isolated. Samples were analyzed via ELISA for OVA-specific IgE according to the manufacturer’s instructions (AbD Serotec, Raleigh, USA). In some experiments, sera were analyzed via ELISA for total IgE and performed according to manufacturer’s instructions (Innovative Research Inc, Florida, USA).

### Flow cytometric analysis of conjunctiva

This procedure has been described elsewhere [17]. Briefly, mice were euthanized 20 min post challenge and surgically procured ipsilateral conjunctivae (i.e. superior and inferior bulbar, fornix, and palpebral regions) were collected. Tissues were digested with 2 mg/ml collagenase type IV and 0.05 mg/ml DNase I (Roche, Basel, Switzerland) for 2 to 3 h at 37°C, as previously described. Suspension were run a through a 70-µm cell strainer and then washed thoroughly. Cells were resuspended in 0.5% BSA buffer, and followed by anti-FcR (CD16/CD32) blockade as per manufacturer’s instructions (BD Pharmingen). Cells were subsequently stained as per manufacturer’s instructions with APC Cy7-conjugated anti-CD45 Ab (clone 30-F11, Biolegend), and PE-conjugated Siglec-F (clone F50-2440, BD Biosciences). Aliquots were made for staining with appropriate isotype controls. All samples were analyzed using a BD LSR II flow cytometer (BD Biosciences).

### Preparation of bone marrow-derived dendritic cells (BMDC)

This procedure has been previously described [17]. Briefly, femurs and tibia were removed from naïve C57BL/6 mice. Bones were flushed by using a syringe filled with RPMI 1640 to release bone marrow (BM) cells. Cells were washed thoroughly and plated (2x10^5^/ml) in RPMI1640 medium supplemented with10% FBS, 1% penicillin/streptomycin, and 20ng/ml mouse GM-CSF (Biolegend). Media was changed on day 4 and loosely adherent cells were collected on day 7.

### In vitro recall stimulation

This procedure is previously described [17]. Briefly, ipsilateral LN (cervical and submandibular) were collected following topical challenge. T cells were enriched for via MACS sorting using anti-CD90.2 Ab according to manufacturer’s instructions (Miltenyi Biotec). Enriched T cells were enumerated via trypan blue exclusion assay, and plated in round-bottom 96-well plate at a concentration of 1.25x10^6^/ml. OVA-pulsed immature BMDCs prepared as described above were plated with T cells at a concentration 0.625x10^6^/ml. In some cultures, LN cells were left unfractionated and subsequently stimulated with 1 mg/ml OVA (without addition of BMDC). All cultures were plated in triplicate wells for up to 24 hr.

### ELISA measurement of T cell cytokines

Cells were plated as indicated above. Following 24 hr OVA stimulation, cultures were restimulated with PMA/ionomycin (Sigma Aldrich) for up to 6 hours. Harvested supernatant was measured via ELISA for IFN-g, IL-4, IL-5, IL-13, IL-10, and TGF-b cytokines, as per manufacturer’s instructions (Ready-set-go ELISA kit, eBioscience, San Diego, USA).

### Intracellular flow cytometry for analyses of T cells in vitro

Cells were plated as indicated above. Following 24 hr OVA stimulation, cultures were restimulated with PMA/ionomycin (Sigma Aldrich) and GolgiStop (BD Biosciences) for up to 6 hours. Cells were collected and stained with PerCP/Cy5.5 conjugated anti-CD4 antibody (cell surface) and stained intracellularly with FITC conjugated anti-IFN-γ, PE/Cy7 conjugated anti-IL-4, PE conjugated anti-IL-5, and APC conjugated with IL-13 antibodies (Biolegend, CA, USA) and were analyzed with a flow cytometer, as described above.

### Flow cytometric analyses of Treg

Following topical challenge, ipsilateral LN (cervical and submandibular) were collected and pooled from freshly euthanized mice. Cells were washed and stained for FITC-conjugated anti-CD4, PE-conjugated anti-CD25, or PE/Cy5 conjugated anti-FoxP3 antibodies (eBioscience). Isotype control was stained with appropriate-matched antibodies (eBioscience). For nuclear staining of FoxP3, a cell fixation/permeabilization kit (eBioscience) was used as per manufacturer’s instruction.

### Treg suppression assay

This procedure was modified from Chauhan et al [18]. Effector T cells (CD4+ CD25-) were MACS sorted from spleens of low-dose (100 µg) OVA sensitized mice. Treg (CD4+ CD25+) were MACS sorted from LN of low- vs. hi-dose sensitized mice post challenge. Treg from immunologically naïve mice were also collected. Sorting was accomplished using the Regulatory T Cell Isolation Kit (Miltenyi Biotec) according to manufacturer’s specification. Co-cultures were established with effector T cells (1 x 10^6^), Treg (5 x 10^4^), OVA pulsed BMDC (1 x 10^5^) in RPMI media including 10% FBS and OVA (1mg/ml) for 3 days in 96-wells. Effector T cell proliferation was evaluated with BrdU incorporation assay according to manufacturer’s instructions (Millipore, MA, USA). Percent Treg suppression = [(proliferation of effector T cells cultured without Treg) – (proliferation of effector T cells cultured with Treg)/ (proliferation of effector T cells cultured without Treg)] x 100.

### Adoptive transfer of Treg

This procedure was modified from Chauhan et al [18]. Sorting was accomplished using the Regulatory T Cell Isolation Kit (Miltenyi Biotec) according to manufacturer’s specification. Treg were MACS sorted from LN of low- vs. hi-dose sensitized mice post challenge. Adoptive transfer of 5 x 10^5^ Treg was performed via intravenous tail vein injection of recipient mice. Recipient mice were sensitized with low-dose intraperitoneal OVA (100 µg) 2 wk prior to adoptive transfer.

### In vivo Treg depletion

CD4+ CD25+ T cells were depleted in vivo via intraperitoneal administration of 1.0 mg of anti-CD25 mAb (PC61; BioXcell, NH, USA) or isotype control Ab (rat anti-mouse IgG1 antibody) 2 d prior to, on the day of, and 7 d after hi-dose OVA sensitization. Depletion of CD4+ CD25+ cells was confirmed in spleen and LNs by flow cytometry.

### Statistical analyses

Graphical data presented throughout are expressed as the mean ± standard error of the mean (SEM). A p< 0.05 was considered statistically significant. Statistical difference was analyzed with the Kruskal-Wallis test, ANOVA or two-tailed student t-test as indicated. Prism software (version 5.0; GraphPad) was used. Normality was tested by Kolmogorov-Smirnov test (Z = 1.166; Asymp. Sig 2-tailed = 0.132).

## Results

### Hi-dose allergen sensitization leads to suppression of allergic immune responses in the mouse model of ocular allergy

**Figure 1:**
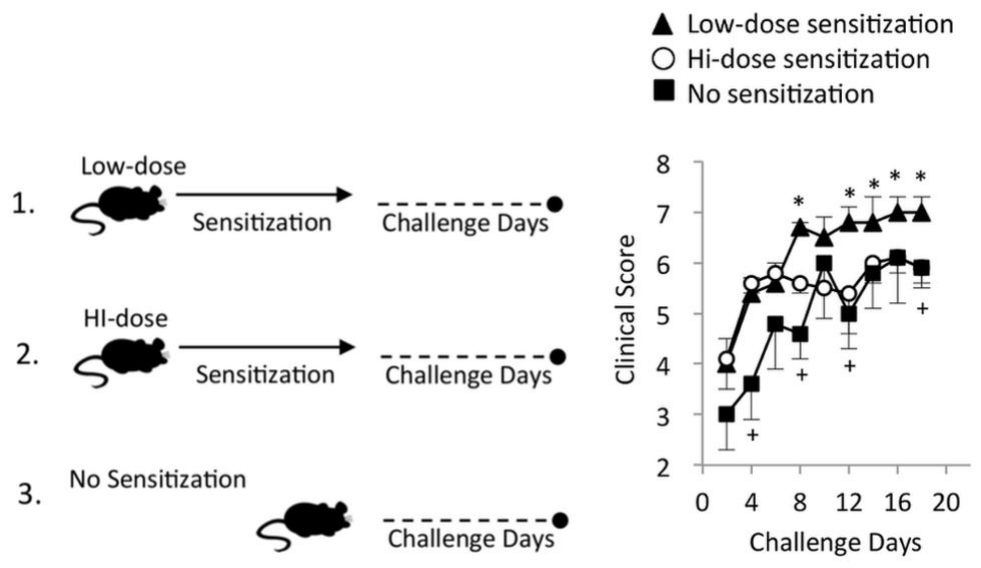
Hi-dose intraperitoneal sensitization suppresses clinical signs of ocular allergy. Mice were intraperitonealy sensitized with OVA/Alum with either hi-dose OVA (1000 µg) or low-dose OVA (100 µg), or left naïve as a control. All mice were rested for 2 weeks and then OVA challenged (including the control) with instillation of OVA eye drops once/d (i.e. Challenge Days) for 18 d. Mice were scored, in a masked fashion, 20 min post challenge for clinical signs (i.e., conjunctival chemosis, hyperemia, lid edema, and tearing/discharge). Data are presented as mean and SEM. Graph shows the results from one experiment (n=4/group), and is representative of 3 independent experiments. Statistical significance was computed via student’s t-test for low-dose sensitization versus no sensitization (^+^ p<0.05), or hi-dose sensitization (*p<0.05).

We first determined whether hi-dose allergen sensitization leads to suppression of allergic immune responses that cause ocular allergy. To test this, we relied on our model previously reported demonstrating robust ocular allergy responses to topical challenges of OVA eye drops in mice that had received intraperitoneal sensitization with low-dose OVA (100 µg OVA) [17]. In the current study, we increased this dose 10-fold, to 1000 µg of OVA, to generate the hi-dose allergen sensitization regimen. Two weeks following hi-dose versus low-dose sensitization, mice were challenged topically with OVA eye drops once daily for more than 18 d (Figure **1**). To assess the effect of dose level on ocular allergy clinical signs, mice were scored in a masked fashion for clinical signs (i.e., conjunctival chemosis, hyperemia, lid edema, and tearing/discharge) 20 min post challenge [17]. Non-sensitized mice were also challenged as a negative control.

Results from this experiment demonstrated a statistically significant (p<0.05) increase for low sensitization over no sensitization controls on Challenge Days 4, 8, 12, 14, 16, and 18 (Figure **1**). This trend was maintained until termination of experiment on day 28 (data not shown). Clinical responses in low-dose sensitization were also statistically greater than those of hi-dose sensitization on Challenge days 8, 12, 14, 16, and 18 (Figure **1**). This trend was also maintained though termination on day 28 (data not shown). Taken together these results indicate that hi-dose OVA sensitization leads to impaired clinical signs as compared to low-dose OVA sensitized mice, relative to levels seen with challenging non-sensitized mice.

We next determined the effect of hi-dose allergen sensitization on allergic immune responses in the ocular allergy model. Thus, hi- versus low-dose sensitized mice were challenged topically and euthanized for collection of conjunctivae, sera, and LN. Conjunctivae were harvested to enumerate eosinophils (CD45+ Siglec-F+) [19] via flow cytometry. Sera were collected to evaluate OVA specific IgE levels via ELISA. Draining LN were collected so that T cells could be stimulated with OVA in vitro for supernatant quantitation of IL-4, IL-5, and IL-13 via ELISA.

**Figure 2:**
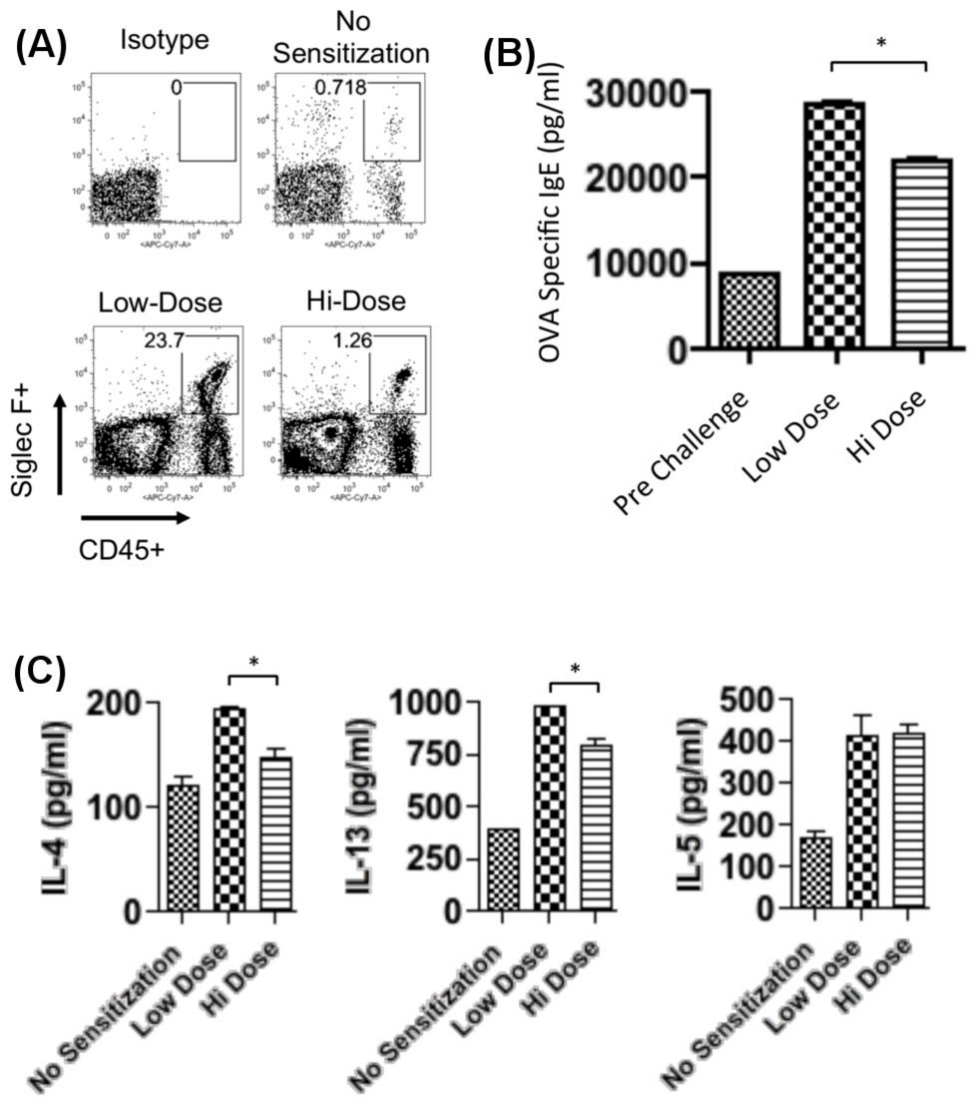
Allergic immune responses are suppressed in response to hi-dose intraperitoneal sensitization. (**A**) Hi-dose intraperitoneal sensitization leads to impaired eosinophil infiltration. Excised conjunctivae of topically challenged eyes were pooled (n=4 mice/group) and prepared into single-cell suspensions for flow cytometric analysis for CD45 and Siglec-F [19]. (B) Hi-dose intraperitoneal sensitization confers impaired OVA specific IgE levels. Sera were collected from topically challenged mice and pooled (n=4 mice/group) to assess OVA specific IgE via ELISA. Data are presented as the mean and SEM (*p<0.05). (**C**) Hi-dose intraperitoneal sensitization suppresses Th2 immune responses. Purified T cells pooled (n=4 mice/group) were stimulated in triplicate wells for 24 h with OVA-pulsed BMDC and then PMA/ionomycin for up to 6 hr. Indicated cytokines in culture supernatants were quantitated by ELISA. Data are presented as the mean and SEM (*p<0.05). (A, B, C) Data presented are from one experiment, which are representative of at least 2 two independent experiments.

For conjunctiva, we observed an approximate 20-fold decrease in the frequency of eosinophils, from 23.7% in low-dose sensitized mice to 1.26% in hi-dose allergen sensitization (Figure **2 a**). Data from repeated experiments are shown in Figure **S1 (a)**. Similarly for the sera, we found a statistically significant reduction in OVA specific IgE in hi-dose allergen sensitized mice, as compared to low-dose sensitization (Figure **2 b**). In addition, T cells from hi-dose sensitized mice displayed reduced IL-4 and IL-13 levels in vitro to OVA stimulation (Figure **2 c**). Taken together, these data indicate that hi-dose allergen sensitization leads to a suppression of allergic immunity and clinical responses in ocular allergy.

### Hi-dose allergen sensitization is associated with increased Treg activity

**Figure 3:**
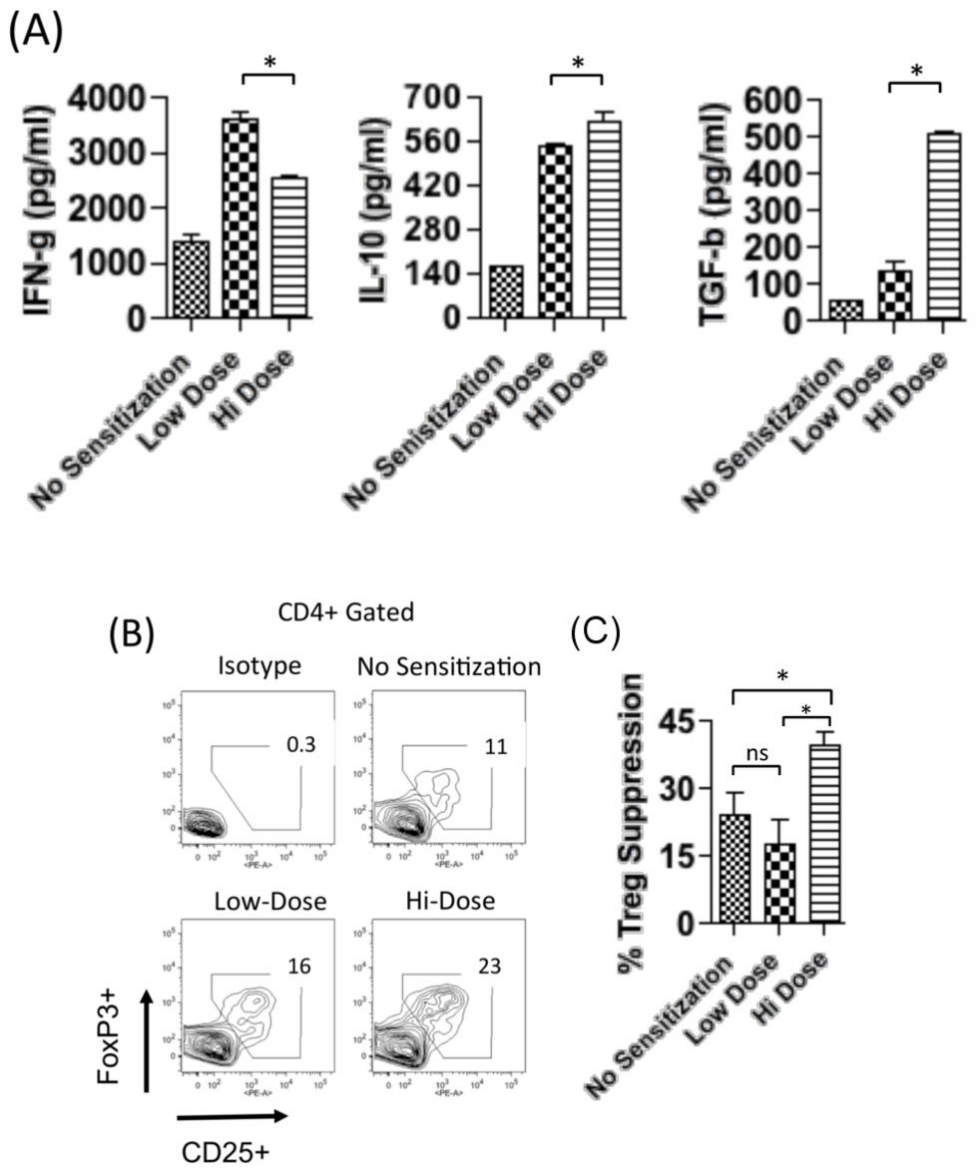
Allergy modulation in hi-dose intraperitoneal sensitization is associated with augmented Treg activity. (**A**) Increased TGF-b expression by T cells in response to hi-dose intraperitoneal sensitization. Purified T cells pooled (n=4 mice/group) were stimulated in triplicate wells for 24 h to OVA-pulsed BMDC and then restimulated with PMA/ionomycin for up to 6 hr. Indicated cytokines in culture supernatants were quantitated by ELISA. Data are presented as the mean and SEM (*p<0.05) (**B**) Hi-dose intraperitoneal sensitization is associated with increased frequencies of Treg (CD4+ CD25+ FoxP3+). Pooled draining LNs (n=4 mice/group) were analyzed via flow cytometry. (**C**) Treg in hi-dose intraperitoneal sensitization confers increased suppression of effector T cell proliferation in vitro. Sorted effector T cells (CD4+ CD25-) from sensitized mice were stimulated in vitro with OVA-pulsed BMDCs. Sorted Treg (CD4+ CD25+) pooled (n=4 mice/group) were added to cultures. Suppression of T cell proliferation by Treg was quantitated by BrdU incorporation assay relative to T cell proliferation in the absence of Treg. Data are presented as the mean and SEM (*p<0.05). (**A**, **B**, **C**) Data presented are from one experiment, which are representative of at least 2 two independent experiments.

Having identified that hi-dose allergen sensitization leads to allergy modulation in ocular allergy, we next set out to begin determining by which mechanism(s) this may occur. Particular cytokine milieus to explain such suppression in ocular allergy can include an IFN-g mediated (i.e., via Th1 polarization), IL-10 mediated (i.e. via Tr1 cells), and/or a TGF-b mediated (i.e., via CD4+ CD25+ FoxP3+ Treg) processes. To examine this, hi- versus low-dose sensitized mice were challenged and LN T cells were stimulated in vitro with OVA for quantitation of culture supernatant IFN-g, IL-10, and TGF-b levels via ELISA. In doing so, we observed that IFN-g levels were significantly decreased in T cells from hi-dose allergen sensitization relative to those from low-dose sensitization (Figure **3 a**). In contrast, we also observed that IL-10 levels were increased in T cells from hi- versus low-dose sensitization (Figure **3 a**). Strikingly, however, we found an approximate 5-fold increase in TGF-b levels in T cells from hi-dose sensitization relative to low dose sensitization (Figure **3 a**). These data suggested a possible role for CD4+ CD25+ FoxP3+ Treg in hi-dose allergen induced suppression of ocular allergy.

Treg frequencies and function were next assessed to examine whether these cells are associated functionally with the allergy modulation in ocular allergy that occurs to hi-dose allergen sensitization. To accomplish this, LN from challenged mice sensitized with low- vs. hi-dose allergen were evaluated via flow cytometry to enumerate Treg frequencies. In doing so, we observed increased frequencies of CD4+ CD25+ FoxP3+ Treg in hi-dose allergen sensitized mice (23%), as compared to low-dose allergen sensitized mice (16%) (Figure **3 b**).

In addition, we determined the effect of hi-dose sensitization on Treg suppressor function, i.e. ability to suppress T cell proliferation [18]. This was accomplished by stimulating proliferation in vitro with OVA-pulsed BMDC of sorted effector T cells (CD4+ CD25-) originating from sensitized mice, and adding sorted Treg (CD4+ CD25+) from hi- versus low-dose sensitized. Treg from naïve mice were also compared as a baseline. Suppression of T cell proliferation by Treg was quantitated by BrdU incorporation assay relative to T cell proliferation in the absence of Treg [18]. Results demonstrated that %Treg suppression from hi-dose sensitized mice was significantly higher than those from naïve mice (**Figure 3 c**); thus, indicating increased Treg suppressor function from hi-dose sensitized mice. In contrast, %Treg suppression from low-dose allergen sensitized mice was not significantly higher than that of Treg from naïve mice (**Figure 3 c**). Taken together, these data indicate elevated Treg activity (i.e. increased frequencies in the LN and increased suppressor function in vitro) is associated with allergy modulation in ocular allergy that occurs to hi-dose allergen sensitization.

### Ablation of Treg during hi-dose sensitization revokes allergy modulation in the ocular allergy model

**Figure 4:**
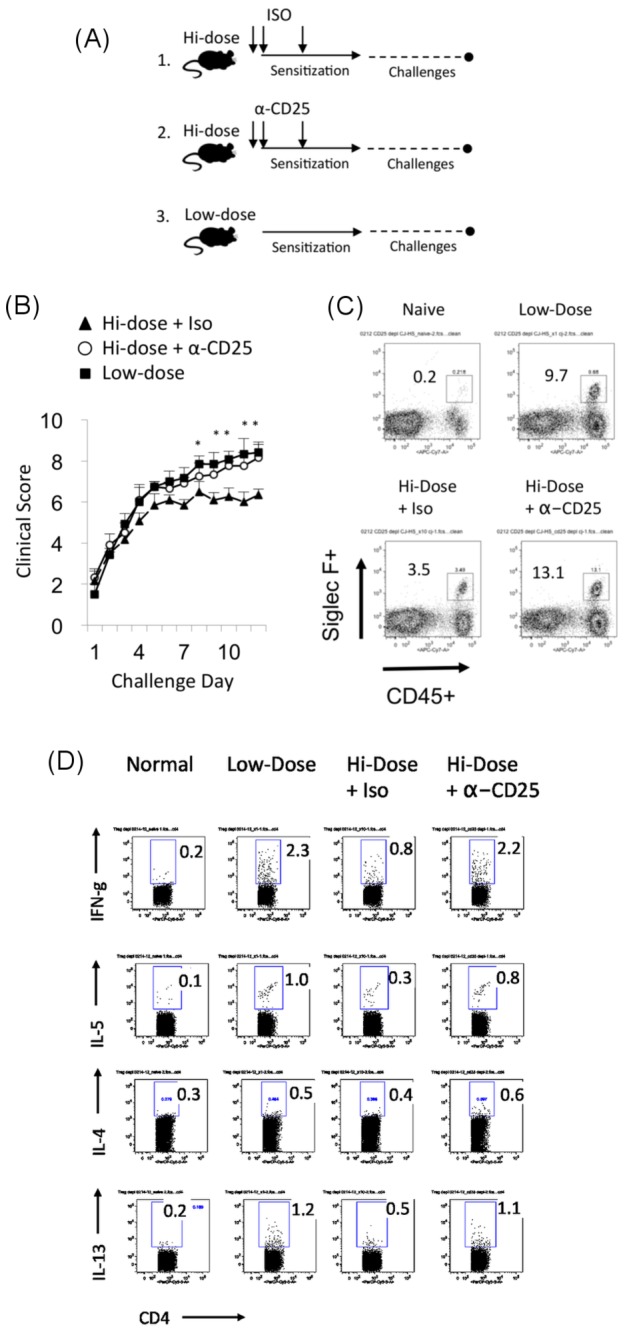
Treg ablation during hi-dose sensitization leads to loss of allergy modulation. (**A**, **B**) Treg were ablated via anti-CD25 Ab (PC61) administration or isotype Ab in hi-dose intraperitoneal sensitized mice and compared to mice that received low-dose intraperitoneal sensitization (n=4mice /group). All mice were challenged once/d and scored for clinical signs in a masked fashion approximately 20 min post challenge. Data are presented as mean and SEM. Statistical significance was computed via student’s t-test for hi-dose sensitized mice treated with isotype vs. anti-CD25 Ab (*p<0.05). (**C**) Suppression of eosinophil infiltration is lost when Treg are ablated during hi-dose intraperitoneal sensitization. Each plot is derived from pooled conjunctivae of an n=4 per group. Data presented is from one experiment, which is representative of two independent experiments. (**D**) Suppression of T cell responses is lost when Treg are ablated during hi-dose intraperitoneal sensitization. LN cells pooled (n=4 mice/group) were restimulated in vitro with OVA for 24 h and then PMA/ionomycin/brefeldin A for up to 6 hours. Flow cytometry was used to quantify frequencies of CD4+ IFN-g+, CD4+ IL-5+, CD4+ IL-4+, and CD4+ IL-5+ T cells. Plots show the results from one experiment (n=4/group), and are representative of 2 independent experiments.

Augmented Treg activity evidenced in association with allergy modulation in ocular allergy that occurs to hi-dose suggested an important role for Treg in this process. To further evaluate this, we determined the effect of Treg ablation during hi-dose allergen sensitization on allergic immune responses and ocular allergy. Treg were ablated by in vivo administration of anti-CD25 Ab blockade (PC61) [20,21] (versus isotype control) in hi-dose sensitization, and we confirmed their resultant absence via flow cytometry of CD4+ CD25+ FoxP3+ (data not shown). Low-dose allergen sensitized mice that did not receive Ab were also included as a control. All mice were challenged once daily and clinically scored in a masked fashion at 20 min post challenge (Figure **4 a**). Interestingly, we observed that the suppression of ocular allergy clinical signs afforded by hi-dose allergen sensitization was lost as a result of Treg ablation, as clinical scores were identical to those seen in low-dose sensitized mice (Figure **4 b**). This was in contrast to hi-dose sensitized mice treated with isotype control Ab, which maintained their modulation as exhibited by significantly lower clinical signs relative to those seen in low-dose sensitized mice (Figure **4 b**).

For eosinophil infiltration, we observed a 3.5% population of eosinophils in hi-dose sensitized mice administered with isotype Ab (Figure **4 c**); whereas eosinophils increased to 13.1% in hi-dose sensitized mice treated with anti-CD25 Ab (Figure **4 c**). The latter was similar to levels seen in low-dose sensitized mice (9.7%) (Figure **4 c**). Data from repeated experiments are shown in Figure **S1 (b)**. Likewise, T cell cytokines to in vitro recall, and conjunctival eosinophil infiltration were examined. We found that hi-dose sensitized mice treated with anti-CD25 Ab showed cytokine levels (i.e., IL-4, 5, 13, and IFN-g) similar to those seen to low dose allergen sensitization (Figure **4 d**); whereas hi-dose sensitized mice treated with the isotype control Ab showed suppressed cytokine levels (Figure **4 d**). T cell data are summarized in graph form in Figure **S1 (c)**.

### Treg from hi-dose allergen sensitized mice adoptively transfers allergy modulation to low-dose allergen sensitized mice

**Figure 5:**
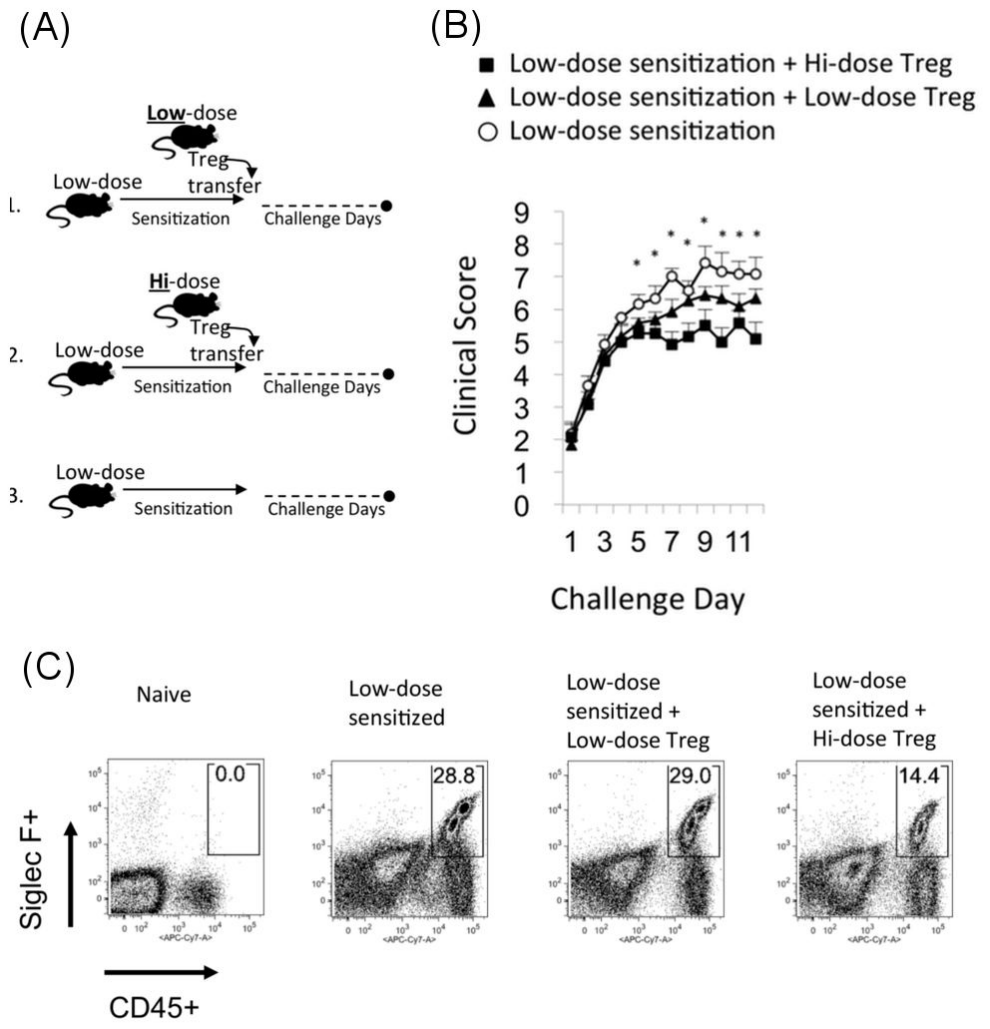
Treg from hi-dose sensitized mice adoptively transfers allergy modulation to low-dose sensitized mice. (**A**, **B**) Low-dose intraperitoneal sensitized mice were adoptively transferred with Treg (5 x 10^5^) from hi-dose versus low-dose intraperitoneal sensitized mice. Low-dose intraperitoneal sensitized mice without adoptive transfer were included as a control. All mice were challenged once/d and scored for clinical signs in a masked fashion approximately 20 min post challenge. Graph shows the results from one experiment (n=4/group), and is representative of 2 independent experiments. Statistical significance was computed via student’s t-test for low-dose sensitized mice transferred with lo-dose vs. hi-dose Treg (*p<0.05). (**C**) Adoptive transfer of Treg from hi-dose intraperitoneal sensitized mice conferred suppression of eosinophil infiltration. Each plot is derived from pooled conjunctivae of an n=4 per group. Data presented is from one experiment, which is representative of two independent experiments.

Having demonstrated evidence for a Treg requirement in ocular allergy modulation arising from hi-dose allergen sensitization, we next set out to determine whether Treg from hi-dose sensitized mice can adoptively transfer suppression to low-dose allergen sensitized mice. To accomplish this, low-dose allergen sensitized mice were adoptively transferred with Treg from hi-dose sensitized mice versus Treg from low-dose sensitized mice (Figure **5 a**). Low-dose sensitized mice not adoptively transferred were also included as a control (Figure **5 a**). All mice were challenged topically once daily and scored clinically in a masked fashion 20 min post challenge. In addition, mice were euthanized and conjunctivae collected for flow cytometric analysis to enumerate eosinophil infiltration. Results from this experiment revealed that adoptive transfer of hi-dose Treg caused a significant suppression of clinical signs relative to low-dose sensitized mice that were not adoptively transferred (Figure **5 b**). This is in contrast to adoptive transfer of Treg from low-dose sensitized mice, which resulted in only a modest to marginal suppression of clinical signs (Figure **5 b**). Data from flow cytometry analysis of conjunctiva showed that adoptive transfer of Treg from hi-dose sensitized mice led to impairment in eosinophil infiltration (14.4%) as compared to mice that received Treg from low-dose sensitized mice (29.0%) (Figure **5 c**). Taken together, these data therefore indicate that Treg from hi-dose allergen sensitized mice adoptively transfers hi-dose allergen induced modulation to low-dose allergen sensitized mice.

## Discussion

Modulation of Th2 in allergy to hi-dose intraperitoneal sensitization has been previously appreciated, albeit shown here for the first time to also be relevant in the murine ocular allergy model. However, until now, a role for CD4+ CD25+ FoxP3+ Treg has not been shown. Allergy modulation to hi-dose intraperitoneal sensitization was previously hypothesized to occur as a mere secondary effect of priming a Th1-dominant response—which can in turn suppress other Th subsets such as Th2 [6-8]. However, Th1 polarization cannot fully explain this effect. Indeed, other T helper lymphocyte subsets, i.e. Th1 and Th17, are widely appreciated to also play an important role in allergy immunopathogenesis [13-16]—although the role of Th17/IL-17, if any, in ocular allergy remains unclear [23]. Furthermore, we present strong evidence that indicate a central role for Treg (CD4+ CD25+ FoxP3+) in mediating allergy modulation in ocular allergy to hi-dose intraperitoneal sensitization. Evidence to support this includes our finding that Treg ablation revokes hi-dose sensitization induced modulation in ocular allergy, and we also show that Treg from hi-dose sensitized mice adoptively transfers this modulatory effect. Thus, given the central role of Treg that was revealed in this study, we have concluded that allergy modulation in ocular allergy arising from hi-dose intraperitoneal sensitization is principally a Treg mediated tolerance.

The first clue that implicated this was our initial findings demonstrating that hi-dose intraperitoneal sensitization leads to impaired ocular allergy clinical signs. This was associated with decreased eosinophil recruitment, decreased Th2 cytokines to recall in vitro stimulation, and reduction in sera IgE levels. Consistent with our findings, Aguilar-Pimentel et al and others have shown that hi-dose intraperitoneal sensitization leads to modulation of Th2 in the allergic asthma model [12]. Data herein suggest that this effect in our model was not associated with a dominant Th1 response, as hi-dose sensitization was found here to have suppressed IFN-g levels indicated by recall stimulation of LN T cells in vitro. This is in line with work by Sakai et al (as well as by Morakata et al) in the allergic asthma model, who reported suppressed IFN-g levels to in vitro recall stimulation of splenocytes from hi-dose (1000 µg OVA) allergen sensitized mice [10,11]. It is noteworthy, however, that both Sakai and Morakata et al found that IFN-g levels were not suppressed in lung homogenate or bronchial lavage of hi-dose sensitized mice [10,11]. While the reasons for this are not clear, the current study has revealed multiple lines of evidence indicative of a central Treg role being principally responsible for allergy modulation in hi-dose intraperitoneal sensitization.

Initial direct evidence to suggest a central role for CD4+ CD25+ FoxP3+ Treg in this modulatory response was our identification of very high expression levels of TGF-b by T cells to recall allergen stimulation in vitro. We observed a near 5-fold increase in TGF-b levels in T cells from hi-dose as compared to low-dose sensitized mice, whereas the increase in IL-10 levels was less than 0.25-fold.

Furthermore, we also observed increased CD4+ CD25+ FoxP3+ Treg frequencies in the LN, which suggests expansion of inducible Treg corresponding with previous reports [24,25]. Interestingly, our findings point to the possibility that the increased Treg frequencies may not be solely responsible for modulation, as increased suppressor function of Treg from hi-dose sensitized mice was also observed here. Thus, hi-dose allergen sensitization augments Treg activity via increased suppressor function and expansion of Treg. This is somewhat different to CD4+ CD25+ FoxP3+ Treg that mediate Th1 modulation in promotion of corneal transplant survival, which is associated with increased suppressor function, but not increased Treg frequencies [18].

Data from our Treg ablation studies further supported the conclusion that Treg play a central role in allergy modulation arising from hi-dose intraperitoneal sensitization. Treg ablation was performed via administration of anti-CD25 Ab (PC61) blockade [20-22] 2 d prior to, on the day of, and 7 d post administration of hi-dose allergen immunization—a regimen which we confirmed to systemically ablate Treg (data not shown). Results of this experiment showed that allergy modulation was lost when Treg were ablated during hi-dose allergen sensitization. Increased clinical scores, eosinophil recruitment, and IL-4, -5, -13 and IFN-g cytokine levels to recall stimulation, indicated this. Our findings are in line with Boudousquié et al, demonstrating anti-CD25 Ab ablation of Treg impairs modulation arising from intranasal allergen treatment in the allergic asthma model [24]. Relatedly, Leech et al reported that Treg ablation using anti-CD25 Ab blockade in intraperitoneal sensitized mice led to impaired allergic asthma resolution [26].

Perhaps the strongest line of evidence in the current study to implicate a central Treg role in allergy modulation in ocular allergy arising from hi-dose intraperitoneal sensitization is the adoptive transfer experiments performed. Indeed, numerous converging reports have already demonstrated protection from allergic immune responses via adoptive transfer of Treg from naïve mice [24-27]. Interestingly, we found that modulation of Th2 responses in low-dose sensitized mice was adoptively transferred with Treg harvested from hi-dose allergen sensitized mice—but only marginally so to those from low-dose sensitized mice. These data demonstrate that it is not simply the action of Treg adoptive transfer in our model that mediates fulminant modulation, but rather it is the Treg induced by hi-dose intraperitoneal sensitization that mediates this effect.

Collectively, we show for the first time that Treg are central to allergy modulation arising from hi-dose intraperitoneal sensitization, and demonstrated this in the murine model of ocular allergy [17,28,29]. We found that hi-dose allergen sensitization leads to impairment of allergic immune responses and ocular allergy clinical signs. Treg ablation revokes hi dose sensitization-induced modulation, and Treg from hi-dose sensitized mice effectively transfers this modulatory effect to low-dose sensitized mice. Thus, we conclude that allergy modulation arising from hi-dose intraperitoneal sensitization is principally a Treg-mediated tolerance. Thus, our findings elucidate the mechanism by which hi-dose intraperitoneal sensitization leads to allergy modulation. Furthermore, these results promote the future use of hi-dose intraperitoneal sensitization to further elucidate the role of tolerance and Treg in allergy modulation.

## Supporting Information

Figure S1 (**A**) Experimental repeats from data depicted in Figure 2a. Eosinophil quantitation by flow cytometry analysis of CD45+ Siglec F+ cells from conjunctivae of the indicated groups. (**B**) Experimental repeats from data depicted in Figure **4c**. (**C**) Flow cytometry data of experimental repeats depicted in Figure **4d**. Y-axes are %cytokine+ (e.g. IFN-g) of CD4+ T cells.
(PDF)
