# Supplementary figures and images for: Ocular Allergy Modulation to Hi-Dose Antigen Sensitization Is a Treg-Dependent Process
Source: PLoS One. 2013 Sep 27;8(9):e75769. doi: 10.1371/journal.pone.0075769 (PMC3785421; doi:10.1371/journal.pone.0075769)

(A)

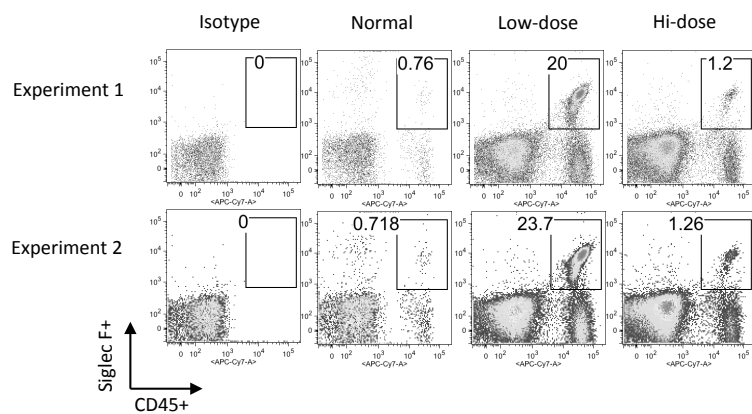

(B)

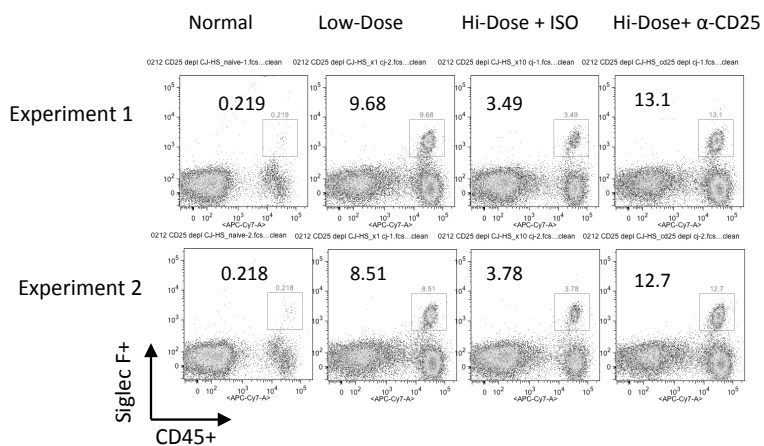

(C)

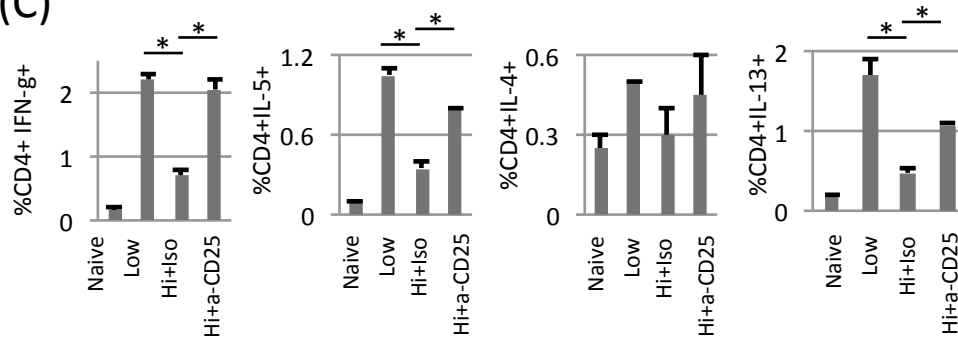

Supplement: Figure S1 — (A) Experimental repeats from data depicted in Figure 2a. Eosinophil quantitation by flow cytometry analysis of CD45+ Siglec F+ cells from conjunctivae of the indicated groups. (B) Experimental repeats from data depicted in Figure 4c. (C) Flow cytometry data of experimental repeats depicted in Figure 4d. Y-axes are %cytokine+ (e.g. IFN-g) of CD4+ T cells. (PDF) [file pone.0075769.s001.pdf]
